# Acceptability of short text messages to support treatment adherence among adolescents living with HIV in a rural and urban clinic in KwaZulu-Natal

**DOI:** 10.4102/sajhivmed.v20i1.976

**Published:** 2019-10-03

**Authors:** Mthokozisi A. Cele, Moherndran Archary

**Affiliations:** 1 Department of Paediatrics and Child Health, University of KwaZulu-Natal, Durban, South Africa

**Keywords:** adolescents on antiretroviral therapy, HIV, text messaging system, adherence support, retention cell phone technology, mHealth

## Abstract

**Background:**

The use of mobile communication technologies (mHealth) has improved adherence and viral suppression among HIV-infected adults. Adolescents have disproportionally lower levels of adherence and viral suppression compared with adults, potentially impacting the goal of 90% viral suppression by 2030.

**Objectives:**

The aim of this study was to evaluate the acceptability of using short message service (SMS)-based mHealth interventions as a tool to improve adherence in HIV-infected adolescents in a rural and urban clinic in KwaZulu-Natal (KZN).

**Method:**

A cross-sectional study with a sample size of 100 participants was conducted in a rural and urban clinic in KZN, from January 2018 to June 2019. Fifty participants were sequentially enrolled from each clinic. A questionnaire was self-administered with the assistance of the treating clinician depending on the adolescent’s level of understanding. Informed consent was obtained from guardians and questionnaires were anonymised. Appropriate descriptive and comparative statistics were used.

**Results:**

The mean age of participants was 15 years, with 88% having access to a mobile device (MOD). There was no significant difference in MOD ownership between rural and urban participants. Majority of participants (65%) were willing to receive SMS-based adherence support with no difference between rural and urban area.

**Conclusion:**

With high rates of MOD ownership and acceptability (willingness to use mHealth to improve health status), SMS-based mHealth interventions have the potential to improve adherence and viral suppression in adolescents living with HIV in both rural and urban KZN. Further studies with a larger sample size need to be conducted to further explore these findings.

## Introduction

In 2015, an estimated 1.8 million adolescents worldwide were living with HIV,^1^ with over two-thirds living in sub-Saharan Africa (SSA).^2^ In 2018, an estimated 310 000 adolescents were living with HIV in South Africa.^3^ The earlier initiation of antiretroviral treatment (ART) in vertically HIV-infected children has resulted in more children surviving into adolescence potentially increasing these estimates in the future. With the aim of controlling the HIV epidemic, the World Health Organization (WHO) launched the 90-90-90 campaign, which aims for 90% of people living with HIV knowing their HIV status, 90% started on ART and 90% with a suppressed HIV viral load.^4^

One of the challenges in achieving the 90-90-90 targets is the poor adherence among adolescents in Africa.^5^ Poor adherence is associated with lower rates of viral suppression both in adult and adolescent populations.^6,7,8^ A recent systemic review estimated that viral suppression ranged from 27% to 89% in Africa.^9^ West and Central Africa had the lowest viral suppression rate (8%), while East Africa had the highest (56%).^10^ In South Africa, a study conducted in Gauteng and Mpumalanga found that adolescents were more likely to be unsuppressed by 12 months (Risk Ratio 2.30, 95% CI, 1.38–3.82) than adult patients.^11^

Adolescents also have lower rates of retention in care compared with adults.^12^ An evaluation of lost to follow-up in adolescents starting ART in four SSA countries was 20% at 3 months and 44% at 12 months.^13^ In a study conducted in a cohort of 241 adolescents in South Africa between 2007 and 2015, 89% were retained in care and 81% achieved viral suppression. Furthermore, adolescents attending designated adolescent clinics had higher retention rates (95%) compared with those attending paediatric clinics (85%). The needs of adolescents should be addressed differently from the general population,^14^ with individualised care associated with higher adolescent retention in care.^15^ Mobile communication technologies in medical care (mHealth) have the potential to address some of these issues.

mHealth refers to the use of mobile devices (MODs), such as mobile phones to support practice of medicine and public health.^16^ There are several variations to mHealth interventions, including short messaging service (SMS) reminders, gaming applications (apps) and interactive mobile apps. A mobile app study conducted in New England found improved motivation in taking antiretroviral therapy,^17^ while a MedMinder pill counter that generates automated SMS to adolescents found that there was a significant increase in CD4 counts.^18^

A randomised controlled trial of a personalised SMS reminder to promote adherence in adults in Kenya improved adherence from 28% to 64% after 3 months.^19^ In studies conducted in Uganda and Kenya found that 97% of the surveyed participants thought that SMS reminders would improve treatment adherence.^19,20^ A study conducted in Ethembeni clinic in Durban found that 98% of the adult participants remembered their medications and would recommend the service to a friend.^21^ Concerns expressed regarding mHealth included privacy and security with unintentional sharing of personal information being of most concern.^22^ Sharing of MODs is common in Africa and has implications on mHealth interventions. The majority (88%) of respondents from a study in Mozambique felt that unauthorised access to text messages could result in accidental disclosure of their HIV status because they share MODs.^23^ Sharing of MODs may also result in a delay in relaying of the message or message not being delivered to right person.^20^ Intermittent and limited funding for public health and sustainable mHealth interventions is a major problem especially in resource-limited countries with competing priorities.^24^

The majority of mHealth studies have been based on an SMS reminder that is sent as daily or weekly reminders. More research is needed to determine the optimal frequency of messages to support adherence. Improvements in mobile technology have allowed interactive text messages that have been shown to help with emotional support and build patient knowledge and allowed the adolescents to ask questions.^7^ However, SMS provides an easy, cheap and accessible technology for mHealth that is accessible even in remote areas with limited resources.^25^ An mHealth intervention (MomConnect) in pregnant women was launched in South Africa in 2014 and has been successfully implemented in the adult population. To our knowledge, there is limited data evaluating mHealth interventions in adolescent populations in KwaZulu-Natal (KZN), especially comparing rural and urban populations.

The aim of our study was to evaluate the acceptability of using SMS-based mHealth interventions as the tool for mHealth in adolescents living with HIV from KZN, comparing a rural and urban clinic, with the aim to support adherence and retention in care. Furthermore, we explored the preferred type of mHealth support (peer support or daily or weekly reminders).

## Methodology

We conducted a cross-sectional observational survey in a rural and urban clinic in KZN from January 2018 to June 2019. The two study sites, a rural clinic (Umbumbulu clinic) and an urban clinic (King Edward VIII Hospital [KEH]) were selected based on the geographic location and patient profile. Umbumbulu clinic is in a deep rural area in a sub-district of KZN, 30 km south of Durban. The Umbumbulu clinic is a primary healthcare centre serving the marginalised community of Umbumbulu, with a high rate of unemployment, poor transport system and patients have to walk 3 km – 5 km to access the clinic. Umbumbulu clinic is a combined service serving children, adolescents and adults. The ARV clinic (Philani clinic) at KEH, Durban, serves an urban and peri-urban area, including patients from formal and informal housing in Umbilo, Cato Manor, Manor Gardens and Mayville. These communities include low- and middle-class families.

### Population and sample

We sequentially enrolled 100 adolescents (12–19 years of age) living with HIV on ART from the adolescent clinics in the two sites. As an exploratory pilot study, the sample size of 100 was based on availability of limited resources and the patient numbers at each of the study sites. The sample size was powered to detect a difference in mHealth acceptability of 70% between the rural and urban sites with a power of 95% and alpha of 0.05. A further study with a larger sample size would need to be performed to determine smaller differences between the two groups. Fifty participants were enrolled from each clinic with an equal distribution of males and females. Informed consent to participate in the study was provided by the parent or guardian (telephonic consent was provided when the adolescent came alone), the adolescent provided signed assent and those above 18 years signed informed consent. S.N. at Umbumbulu Clinic and C.K. at KEH recruited participants while registering their details before the doctor consultation. No patient incentives were provided for participation in the study as patients were recruited during scheduled visits and the questionnaire was completed while waiting for their consultations. The investigator conducted a 2-h workshop 2 months prior to the commencement of the study at both clinics, to engage with S.N. and C.K. and standardise the survey collection. Participants were provided with a pen and questionnaire in the waiting area and answered the questionnaire while waiting for their consultation. After completion, the questionnaire was dropped in the box inside the consultation room.

An anonymous questionnaire was used as a tool to collect data from the participants. The questions were divided in 10 sections, mainly containing information about age, language frequently used, sex, gender, level of education, MOD ownership and perceptions about SMS mHealth interventions. The questionnaire was available in IsiZulu and English. The questionnaire had been piloted at KEH as part of the Passages project.

### Statistical analysis

Results were reported as means for continuous variables and as frequencies and percentages for categorical variables. Chi-square tests for categorical data and Student *t*-tests for continuous data were used to compare the difference in covariates between the rural and urban clinic sites.

A chi-square test was also used to assess phone ownership and mobile health technology feasibility by level of education. If more than 25% of the cells had expected counts less than five, a Fisher’s exact test was run in place of the chi-square test. This is necessary because having small expected counts violates an assumption of the chi-square test. Analysis was performed using SAS 9.4.

### Ethical considerations

Approval for this study was obtained from the University of KwaZulu-Natal Biomedical Research Ethics Committee (BREC Number BE480-17), the KZN Department of Health (KZ_201801_031) and the two institutions.

## Results

The key demographics of this survey from both rural and urban areas are depicted in Table 1. The mean age was 15 years (range 13–19 years). There was an almost equal distribution between males and females as per the study design. The majority of the population in this sample was black Africans and the preferred language of communication was IsiZulu. There were significant differences between the participants in the urban and rural clinics with regard to age and level of education.

**TABLE 1:** Demographic characteristics of the study population by clinic site (*N* = 100).

Covariates	Total (*N* = 100)	Rural (*N* = 50)		Urban (*N* = 50)		*p*
	*N*	*N*	%	*N*	%	
Age (mean)	15.98	16.8	-	15.2	-	0.0002*
**Sex**						
Male	49	23	46	26	52	0.6859†
Female	43	22	44	21	42	-
Unclassified	8	5	10	3	6	-
**Population group**						
Black	97	50	100	47	94	0.1775‡
Mixed race	3	0	0	3	6	-
**Primary language**						
Zulu	97	50	100	3	6	0.2424‡
English	3	0	0	47	94	-
**Education**						
No education	1	1	2	0	0	0.0049‡
Primary	15	6	12	9	18	-
Secondary	50	19	38	31	62	-
Tertiary	17	14	28	3	6	-
Unclassified	17	10	20	7	14	-

* , *p*-value reflects results of *t*-test.

† , *p*-value reflects results of Chi-square test.

‡ , *p*-value reflects results of Fisher’s exact test. More than 25% of cells had expected counts less than 5 making Chi-square test inappropriate.

Access to an MOD was high as shown in Table 2, with 88% of adolescents surveyed having access to an MOD. There was no statistical difference in MOD ownership between rural and urban sites (92% vs. 84%, *p* = 0.2). The MOD ownership was almost the same between males and females (88% vs. 83% *p* = 0.19). There was a high frequency of SMS messages use in the rural site as compared to urban (40% vs. 26%, *p* = 0.08). Despite high MOD ownership, 48% of participants in the urban site had never sent an SMS text message. The majority of the study population at both sites had not sent or received any form of text message from a medical provider; however, adolescents at both the rural and urban sites were willing to use mobile technology to access healthcare (Table 2).

**TABLE 2:** Feasibility of mobile health technology by clinic site (*N* = 100).

Covariates	Rural (*N* = 50)		Urban (*N* = 50)		*P*
	*N*	%	*N*	%	
**Phone Ownership**					
Yes	46	92	42	84	0.2184
No	4	8	8	16	
**SMS Message Frequency**					
Every day	20	40	13	16	0.0802*
Every 2-6 days	3	6	5	10	
Once a week	5	10	1	2	
Once every 2 weeks	4	8	1	2	
Never	15	30	24	48	
**Text Language**					
Zulu	37	74	25	50	0.1057
English	13	26	18	36	
**Have you ever sent a SMS to medical provider?**					
Yes	5	10	5	10	0.7992*
No	44	88	36	72	
Do not know	1	2	2	4	
**Have you ever received a SMS from medical provider?**					
Yes	9	18	5	10	0.5111*
No	37	74	33	66	
Do not know	3	6	5	10	
**Would you be willing to receive an SMS from a medical provider in the future?**					
Yes	31	62	33	66	0.1226
No	11	22	3	6	
Maybe	8	16	7	14	

* , *p*-value reflects results of Fisher’s Exact Test. More than 25% of cells had expected counts less than 5 making Chi-Square Test inappropriate.

Feasibility of mHealth interventions was stratified by participant level of education (Table 3). Fifty per cent of the study population was attending secondary school with a statistically significant increase in MOD ownership with higher level of education (*p* = 0.0016). Only 38% of participants reported that they use SMS every day; however, 34 % reported that they never sent an SMS text message. Use of mHealth was feasible particularly in adolescents who attend secondary school with high levels of MOD ownership and 67% willing to receive SMS regarding their health.

**TABLE 3:** Feasibility of mobile health technology by education level (*N* = 83).

Covariates	No Education (*N* = 1)	Primary (*N* = 15)	Secondary (*N* = 50)	Tertiary (*N* = 17)	*P*
**Phone Ownership**					
Yes	0	11	49	15	0.0016*
No	1	4	1	2	
**SMS Message Frequency**					
Every day	0	5	19	4	0.7902*
Every 2-6 days	0	1	6	1	
Once a week	0	0	2	2	
Once every 2 weeks	0	0	4	1	
Never	1	8	17	8	
**Text Language**					
Zulu	1	11	27	11	0.6543*
English	0	4	19	4	
**Have you ever sent a SMS message to medical provider?**					
Yes	1	1	3	1	0.2200*
No	0	13	42	14	
Do not know	0	1	2	0	
**Have you ever received a SMS message from medical provider?**					
Yes	0	2	4	5	0.2252*
No	1	9	39	11	
Do not know	0	2	4	0	
**Would you be willing to receive an SMS message from a medical provider in the future?**					
Yes	0	11	33	9	0.4181*
No	1	1	7	4	
Maybe	0	2	7	3	

* , *p*-value reflects results of Fisher’s Exact Test. More than 25% of cells had expected counts less than 5 making Chi-Square Test inappropriate.

## Discussion

In this exploratory pilot study evaluating the acceptability of text messaging support for adolescents in an urban and rural clinic, we found no significant difference in MOD ownership and acceptability of mHealth between the two sites. Participants in both sites had high MOD ownership, with more than 90% of adolescents in this cohort owning a MOD. There are similar trends in MOD ownership that have been noted in other studies from Africa. A study of MOD ownership among the youth population in Malawi, Ghana and South Africa showed that 88% of youth owned an MOD.^26^ Despite the high levels of MOD ownership, there is a possibility that adolescents share their MODs with other family members, which increased the risks of accidental disclosures of confidential information regarding their health status.^16^ Investing in improving SMS-based mHealth interventions that do not divulge patient information and protecting individual privacy is urgently needed.^16,27^ While there have been different mHealth interventions studied in adult populations in developing and developed countries, these findings support the exploration of mHealth in adolescents residing in both rural and urban KZN.

Telecommunication technologies are emerging as an important means of extending healthcare to patients with limited direct access to a healthcare facility, with a Cochrane review showing that there is growing interest in the use of cell phone technology.^28^ In this study, 65% of adolescents were willing to participate in a mHealth intervention. There are different communication tools that have been used for delivering mHealth, ranging from simple, for example SMS, to more complicated, for example interactive apps. Participants from both clinics found that communicating with a healthcare provider using SMS text messages was an acceptable tool. One of the major potential benefits of mHealth is in improving adherence and retention to care. A study conducted in a rural area of Kenya found that an SMS-based mHealth intervention was associated with 90% improvement in adherence over 48 weeks.^29^ Additional benefits include decreased need for regular follow-up appointments resulting in a positive financial effect for patients as most clinics are far from the community.^18^ Several studies have found a correlation between high usage of mHealth and improvement of adherence.^22,30,31^ The high MOD ownership and willingness to access mHealth at both sites indicate that there is a space for mHealth interventions among these communities.

The higher usage of SMS text messaging in the participants from the rural clinic compared to the urban clinic likely highlights the influence of different access to telecommunication technologies between the settings. The ready availability of Internet-based messaging (e.g. WhatsApp) in urban centres may account for the lower use of SMS text messaging. This finding highlights the need for the use of simple technologies that can be accessed by both urban and rural communities when designing mHealth interventions. Implementation of telecommunication technologies requires adequate infrastructure, sustained budget for operational costs and this will need involvement of public and private sector support for it to be sustainable.^32^

The frequency of preferred messages differs by country, with patients in developing countries preferring to receive messages once a week,^20,29,30^ compared to daily text messaging that was preferred by patients in some developed countries.^33^ This preference may be related to socio-economic status with adolescents in developing countries being more likely to share MODs and less frequent messages would decrease the chances of accidental disclosure. Other suggestions by youth to improve confidentiality of mHealth include the use of MODs with access codes to increase security.^22^ In addition, daily SMS might be intrusive, produce habituation and response fatigue.^34^ There is suggestion that a two-way text messaging may be better than a simple text message reminder. More evidence is warranted regarding the optimal frequency of messaging.^35^ The majority of adolescents attending school may have restriction placed on the use of MOD both at school and at home during the school terms; this is important for the timing of SMS.

While the frequency of messaging is high among adolescents, only 12% of adolescents use cell phones for health-related information^26^; this is lower than previously thought. A study conducted in Malawi, Ghana and South Africa showed that 29% use cell phone technology for health-related issues.^26^ The possible reasons that can be extrapolated for the low usage include that people struggle to differentiate between good and bad information from the Internet about health and the prohibitively high cost of using data.^26^ Text messaging and phone calls maybe unaffordable in certain very low resource settings.^24^ These challenges will need to be addressed to achieve adherence and retention. Our study indicates that mHealth interventions hold promise, as 65% of adolescents are willing to use cell phone technology for health-related information.

Providing healthcare-related messages in a language that the recipient is comfortable with is very important. There are differences in languages used at home between participants in the urban and rural clinic, highlighting the need for the language to be customised to accommodate different ethnic and linguistic groups.

The average age of the participants in this survey is 15 years, with the majority in secondary school. However, if mHealth interventions are to be implemented, then content of messages might need to be different to accommodate different age groups. These messages need to be individualised and free of abbreviations like HIV/AIDS to decrease risk of accidental disclosure.^35^

A limitation of this study was the small sample size, which limited the power of the study to detect smaller differences between the two populations. However, this analysis was aimed at exploring mHealth in rural and urban areas in KZN and provides a rationale for further research in this area. Bias may arise as caregivers consented for enrolment into this survey, which may influence the responses, but assent was obtained and those adolescents who refused participation were not included. Further participants may have shared information while waiting outside the consultation room, which can lead to contamination of the data.

Strategies like mHealth are in line with the Fourth Industrial Revolution, as technology has become the focus. With dwindling international funding for provision of ART globally, this is a key opportunity to use innovative technologies (such as mHealth) to support a sustainable ART programme especially in vulnerable groups.^36^ However, as many African countries struggle with poverty, high unemployment rate and weak economic growth, it will be challenging to channel resources for implementation of such projects.^24^

## Conclusion

This study has demonstrated that adolescents in this study population were willing to use simple SMS technology to communicate their health status with the health system both in urban and rural areas. This demonstrates the acceptability of potential of mHealth interventions that might have positive impact in supporting adherence and retention in care. Further studies are required to identify the most effective types of messaging, cost-effectiveness and sustainability especially in developing countries. This will require partnership with the private sector cellular phone networks while tailoring the intervention to the needs and information required by adolescents.
